# The Cost of Potentially Inappropriate Medications in Nursing Homes in West Occitanie

**DOI:** 10.3390/pharmacy8010039

**Published:** 2020-03-11

**Authors:** Marie Caucat, Alice Zacarin, Vanessa Rousseau, Jean-Louis Montastruc, Haleh Bagheri

**Affiliations:** Department of Medical and Clinical Pharmacology, Centre for PharmacoVigilance, PharmacoEpidemiology and Information on Medications, Faculty of Medicine, INSERM UMR 1027, University Hospital and Faculty of Medicine, 31000 Toulouse, France; marie.caucat@yahoo.fr (M.C.); rousseau.v@chu-toulouse.fr (V.R.); jean-louis.montastruc@univ-tlse3.fr (J.-L.M.); haleh.bagheri@univ-tlse3.fr (H.B.)

**Keywords:** potentially inappropriate medication, nursing homes, deprescribing

## Abstract

**Introduction:** As of 2019, people older than 65 years represent 20% of the French population. Despite several guidelines suggesting to avoid potentially inappropriate medication (PIM) use in elderly, the prevalence of their prescription remains high (25%). Furthermore, PIM could lead to preventable adverse drug reactions (ADRs). The main objective of this study was to determine the direct cost of PIM in older persons living in residential care homes for the elderly (nursing homes). A secondary objective was to assess the potential impact of PIM deprescribing on drug-related health care costs. **Methods:** We undertook a multicenter, retrospective study in 19 care homes for the elderly including 1240 residents. The analysis of prescriptions was carried out according to the European EU(7) PIM list. The cost of each drug was estimated according to the French Medication Insurance database. Furthermore, patient’s comorbidities were studied using Charlson’s comorbidity index. In order to estimate the economic impact of PIM, we used the list of alternative appropriate drugs suggested by EU(7) PIM list and French National Health Authority. An incremental cost per patient was calculated by the difference in costs between PIMs and alternative drugs. **Results:** A total of 7768 lines of drug prescriptions were analyzed. The mean age was 87.6 ± 7.6 years. About 70% (n = 872) of residents received more than five drugs. We identified 959 residents (77.3%) with at least one PIM. The mean cost of PIM was 0.58 euros versus 0.48 euros for alternatives. PIM substitution by alternatives led to save 12 centimes/resident/day. The mean cost of prescription with PIM was 2.8 euros per resident per day (28% of the overall cost of prescription). According to these results, more than 25 million euros can be overall saved for aged persons living in nursing homes for the older people in France per year. **Conclusion:** The prevalence of PIMs among the elderly in nursing homes is high and leads to a significant cost. Deprescribing of these medications could decrease both drug misuse and cost of drug prescription. Further research is needed to estimate the overall cost of PIM exposure outcomes, taking into account the ADRs leading to hospitalization.

## 1. Introduction

As of 2019, people older than 65 years represent 20% of the French population [1]. Approximately 10% of subjects >75 years old live in residential care homes for the elderly (nursing homes), and this goes up to 30% for subjects >90 years old [2]. Long-term care expenses for nursing home residents reached almost 10 million euros in France in 2017 [3]. According to the Caisse Nationale de Solidarité pour l’Autonomie (CNSA; French national funding agency for the elderly and handicapped), 37% of nonhospital care expenses recorded in 2017 for nursing home residents relate to the purchase of medications [4].

Polymedication and potentially inappropriate medications (PIM) are risk factors for drug-induced iatrogenesis [5]. Despite national and international recommendations advising the elimination of PIM in the elderly, the prevalence of their prescription remains high [6]. PIM can cause iatrogenic events with serious clinical and economic consequences [7,8], even though these are potentially preventable. The process of improving the quality of medication management helps to achieve a double objective: reducing iatrogenic events and their consequences for elderly people’s health and reducing healthcare costs [9].

The Occitanie region is the second largest region in France. The region’s population is on average older than the French population. The aging index (ratio of the number of inhabitants aged 65 or over per 100 young people under 20) is the fourth highest in France: it is 89.1, versus a national average of 72.2 [10]. Various efforts have been made in the region to improve the care of the elderly. In 2010, the IQUARE project (Impact of QUAlity control on the development of practices and the functional decline of nursing home REsidents) was implemented in the region to optimize the quality of care practices in nursing homes [11,12].

The main objective of our study is to determine the direct costs incurred by PIM in nursing homes. The secondary objective is to measure the potential impact of the proposed deprescription of PIM, in terms of medication costs.

## 2. Method

### 2.1. Study Population

We carried out a retrospective study on 19 nursing homes in the West Occitanie region. We only included nursing homes that have a dispensary in order to ensure we were only looking at establishments using medications with a price fixed at the national level. Nursing homes with in-house pharmacies were excluded from this study.

For each resident, the following socio-demographic and clinical data were collected:

- Age;
- Sex;
- Iso-Resource Group (IRG): IRG measures the degree of independence on a scale of 1 to 6 for an elderly person: IRG 1 corresponds to total dependency;
- Medical history and comorbidities;
- Long-term prescriptions and their dosages. We have excluded prescriptions involving medications indicated in acute pathologies (antibiotic therapies, etc.). We have also excluded adjunctive drugs (e.g., analgesic, antifungal), topical medications (creams, eye drops), laxatives, and all nonreimbursed drugs.

Using the medical histories and comorbidities, we were able to work out the Charlson index [13]. Widely used in clinical trials, the Charlson index is a tool for predicting short-term mortality in elderly patients. This score is calculated by adding the different weights assigned to 19 medical conditions [14]. Adjustment with the patients’ ages results in a weighted score which varies between 1 (for patients aged 50 to 59 years) to 5 (for subjects between 90 and 99 years). Patients with a Charlson score ≥ 5 have more comorbidities and have an 85% risk of mortality at 1 year. This percentage decreases to 12% for patients with a Charlson score of zero [15].

### 2.2. Identification of Potentially Inappropriate Medications

We have used the European EU(7) PIM list [16] to identify PIM. Medications are considered potentially inappropriate when their risk/benefit ratio is unfavorable and there is a safer alternative available, or when their efficacy has not been proven in a given indication [16]. We then carried out a simulated substitution of the PIM with alternatives suggested on the EU(7) PIM list.

However, we did not consider prescriptions that included a PIM in the following two cases:

- Serious pathologies (epilepsy, Parkinson, heart failure, diabetes, etc.) where it is necessary to have the patient’s exhaustive medical file to propose suitable modifications to prescriptions.
- When the EU(7) PIM list does not suggest an alternative as a substitution, we left the PIM as given on the prescription (for example: amiodarone).

Appendix A shows the PIM alternatives proposed during our prescription analysis.

### 2.3. Cost of Medications

We calculated the daily cost of medications (unit price of the medication adjusted according to the dosage) and included only medications reimbursed by health insurance, the price of which is fixed at the national level. This price is available on the website of the national database of medications [17].

### 2.4. Statistical Analysis

We carried out a descriptive analysis of the sociodemographic and clinical variables of the patients. For quantitative variables, the results were presented in the form of mean ± standard deviation (SD). For categorical variables, we calculated the total numbers and percentages.

The tests used were chosen according to the nature of the variables and the size of the samples: the Mann–Whitney Wilcoxon test and the Kruskal–Wallis test for quantitative variables, and the chi-squared test for qualitative variables.

Finally, using a linear regression model we analyzed the correlation between the cost of prescriptions with at least one PIM and the following four variables: sex, IRG, Charlson score and number of PIM per prescription 

Statistical tests were performed using a two-sided alpha significance level of 5%. Data were analyzed using SAS software version 9.4 (SAS Institute, SAS Campus Drive, Cary, NC, USA).

## 3. Results

### 3.1. Study Population

**Figure 1:**
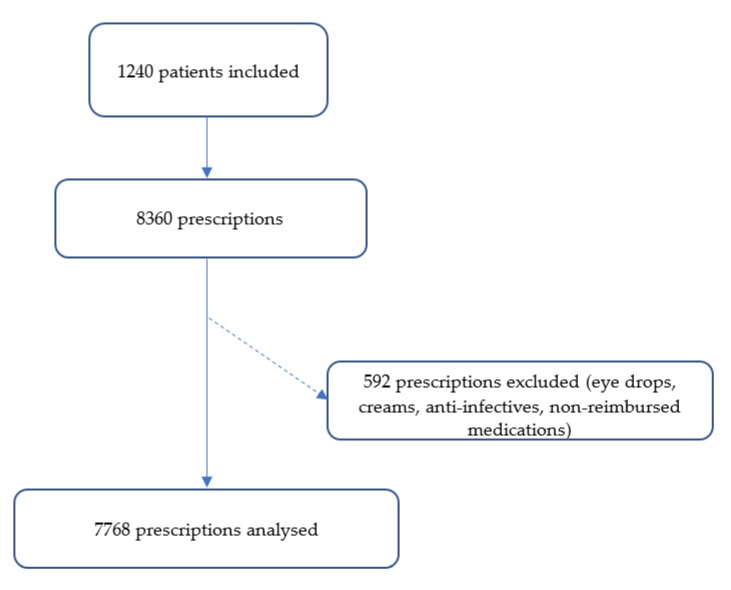
Study flow chart.

A total of 1240 residents were included in our study. After excluding the medications prescribed for an acute condition and those that are not reimbursed, 7768 prescriptions were analyzed (Figure 1). The average age of the population was 87.8 ± 7.6 years: 42.9% (n = 532) of the residents are dependent (IRG 1–2) and only 4.6% (n = 57) of them are independent (IRG 5–6) (Table 1). About 79% (n = 969) of residents have a Charlson score ≥ 5 with an average number of pathologies of 3.9 ± 1.9 (range: 0–13) most frequently comprising hypertension (17.2%, n = 462) and dementia (14.1%, n = 380) (Table 2). The average number of individual medications is 6.2 (±2.9) and 58% (n = 721) of residents take ≥5 medications.

### 3.2. PIM Prescription

**Figure 2:**
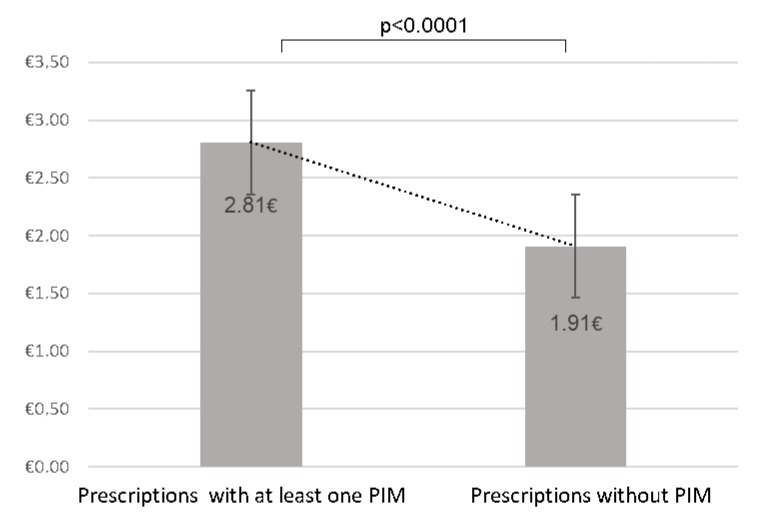
Comparison of the mean cost of prescriptions according to the presence or absence of potentially inappropriate medications.

According to the EU(7) PIM list, 25% (n = 1956) of prescriptions are considered inappropriate (Figure 2): 77% of residents (n = 959) have at least one PIM and the average number of PIMs per resident is 2.04 ± 1.17 (min: 1, max: 9). The percent of patients with more than five drugs is significantly higher when prescriptions contained at least one PIM than those without PIM (*p* < 0.0001).

### 3.3. Cost of Prescriptions and Cost of PIM

The average daily cost of all prescriptions for residents is 2.61 euros. We compared the overall cost of prescriptions without PIM estimated at €1.9 ± 2.7 with that of prescriptions with at least one PIM estimated at €2.81 ± 2.25, showing a statistically significant difference (*p* <0.0001). Furthermore, in prescriptions with at least one PIM, 21% of the cost is incurred by the PIM. The average cost of PIM is €0.58 ± 0.51 per prescription/per day.

Based on a linear regression model, we analyzed the correlation between the cost of prescriptions with at least one PIM and the following four variables: gender, IRG, Charlson score and number of PIM per prescription. Only the number of PIM per prescription is significantly associated with the cost of prescriptions (R^2^ = 0.05, *p* < 0.0001). However, since the variable R^2^ is very small, the number of PIM per prescription is not sufficient in itself to explain the variability of the cost of prescriptions with PIM.

### 3.4. Cost of Prescriptions after Substitution of PIM

**Figure 3:**
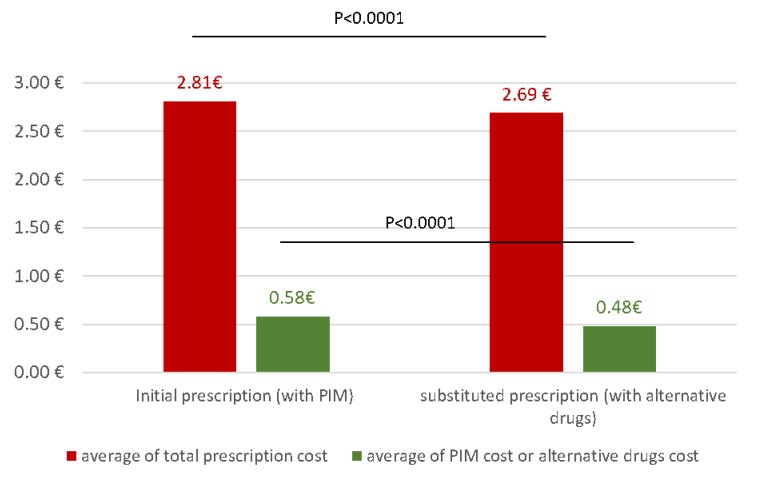
Comparison of total prescription cost between initial and substituted prescriptions (red) and comparison of cost between PIM and alternatives drugs (green).

To analyze the impact of deprescription of PIM on the cost of prescriptions, we substituted the PIM with alternative drugs according to the EU(7) PIM list (Appendix A). Alternative drugs were significantly less expensive than PIM: the average cost of these alternatives was €0.48 ± 0.48, compared to €0.58 ± 0.51 for PIM (*p* < 0.0001) (Figure 3). Consequently, substituted prescriptions cost significantly less than prescriptions with at least one PIM (*p* < 0.0001) (Figure 3). The deprescription of PIM and their substitution with more suitable alternative drugs ensures a significant saving on the daily cost of prescriptions (i.e., €0.12 per day per prescription).

## 4. Discussion

According to the World Health Organization (WHO), polymedication accounts for 4% of avoidable costs caused by the nonoptimal use of medications [18]. Prescription of potentially inappropriate medications is linked to polymedication [19]. In France, the average cost of a prescription is 71 euros for people over 65 [20].

In 2017, 728,000 elderly people in France were living in nursing homes [20]. According to our results, 78% of residents are exposed to PIM, which corresponds to 567,840 residents exposed to PIM in France. If in this study the prescription of PIM and their substitution with more appropriate medications saves 0.12 euros per resident per day, this approach would save more than 25 million euros per year on the national level.

Different studies have looked at the cost of PIM among elderly people in hospitals and as outpatients [21,22,23]. However, very few studies have evaluated the cost of PIM in nursing homes [22]. In the same region, in Occitanie, a recent study carried out at Toulouse University Hospital on 365 elderly hospitalized patients shows that 50% of patients take at least one PIM. This percentage is relatively lower than that of the residents of nursing homes in our study (77%). A study comparable to ours was carried out in 2016 in another French region (Alsace) and shows that PIM are prescribed in 74% of residents [22]. These studies therefore show a difference in the profile of medication use among the elderly living in their own home and those in nursing homes. 

The proper use of medication among elderly people living at home or in nursing homes allows for a reduction in prescriptions of PIM as well as in the cost of the prescription. In our study, the average daily cost of PIM is €0.58 ± 0.51 or 21% of the average cost of prescriptions. This percentage is comparable to the result from hospitals (20%) but significantly higher than the Alsace study (11.6%) for a comparable average daily cost (€0.49 ± 0.76). Alsace has not been part of the PAERPA experiment (Elderly People At Risk of Loss of Autonomy), so we could not compare the indicators of polypharmacy and inappropriate prescription [24] between the two regions. The deprescription of PIM and their substitution results in a significant saving on the cost of prescriptions. According to our results, we can estimate a potential saving of 0.12 euros per resident per day. This figure turns out to be below the savings suggested by a similar study carried out in a hospital environment where the authors suggest savings of around 4 euros per patient [9]. Several factors could explain this difference: the prices of hospital medications are lower than those dispensed to outpatients since these prices are set after an invitation to tender for each active ingredient. Furthermore, the profiles of hospitalized patients and those residing in nursing homes and the sizes of the two samples are different. Finally, Pages et al. [9] weighted the cost according to the length of hospital stay, whereas in our study the cost was estimated at time t.

Geriatric assessment of prescriptions has shown to be effective in improving the quality of life of patients and in reducing adverse events such as fall injuries and readmissions [25].

The strengths of this study are the large sample size and the consideration of the socio-demographic characteristics and comorbidities of the study population. However, we limited our study to the direct cost of PIM and the estimated profit after their substitution without taking into account the overall cost generated by drug-induced iatrogenesis. In fact, one French study estimated the average cost of avoidable hospitalizations linked to iatrogenesis at 9500 euros [26]. In the United States, hospital-related spending on drug-induced iatrogenesis is estimated to be approximately $177 billion [27,28]. In Europe the cost of hospitalizations for falls linked to benzodiazepines is estimated between 1.5 and 2.2 billion euros [28]. Likewise, as mentioned above, we estimated the gross cost at time t, and the duration of exposure to PIM was not taken into account.

In conclusion, PIM are still frequently prescribed to the elderly in nursing homes and generate a significant cost. Deprescribing these drugs improves the quality of medication for this population and reduces the cost of prescriptions. More studies are needed to estimate the overall cost of exposure to PIM by taking into account the duration of exposure to PIM and the adverse drug reactions leading to hospitalization, which generates additional costs.

## Figures and Tables

**Table 1:** Characteristics of the study population.

Characteristics of Residents	Total	Residents with at Least One PIM	Residents without PIM
Number of residents	1240	959	281
Age, mean [± SD]	87.76 [±7.6]	87.36 [±7.7]	89.12 [±6.9]
Sex (%)			
Female	948 (76.5%)	732 (76.3%)	216 (76.9%)
Male	292 (23.6%)	227 (23.7%)	65 (23.1%)
IRG (%)			
1–2	532 (42.9%)	411 (42.9%)	121 (43.1%)
3–4	298 (24%)	229 (23.9%)	69 (24.6%)
5–6	57 (4.6%)	42 (4.4%)	15 (5.3%)
Number of medications, mean [± SD]	6.26 [±2.9]	6.91 [±2.7]	4.07 [±2.2]
Number of medications 0–4	367 (29.6%)	196 (20.4%)	171 (60.9%)
Number of medications 5–9	721 (58.1%)	614 (64%)	107 (38.1%)
Number of medications >9	152 (12.3%)	149 (15.5%)	3 (1.1%)
Charlson index			
0	0	0	0
1–2	22 (2%)	16 (2%)	6 (2%)
3–4	249 (20%)	214 (22%)	35 (12%)
≥5	969 (78%)	729 (76%)	240 (85%)
Number of PIM, mean [±SD]	1.58 [±1.3]	2.04 [±1.2]	0

PIM: Potentially Inappropriate Medication; SD: standard deviation; IRG: Iso-Resource Group.

**Table 2:** Frequency of pathology of the study population.

Pathologies	Number of Patients
Neuropsychiatric	863 (32%)
Dementia	380 (14%)
Depression	245 (9%)
Cerebrovascular disease	103 (4%)
Cardiovascular diseases	763 (28%)
Hypertension	462 (17%)
Atrial fibrillation	135 (5%)
Heart failure	79 (3%)
Metabolic diseases	308 (11%)
Thyroid disease	152 (6%)
Diabetes	125 (5%)
Inflammatory bowel	194 (7%)
Ulcer	98 (4%)
Malnutrition	50 (2%)
Osteoarticular diseases	173 (6%)
Arthrosis	89 (3%)
Osteoporosis	68 (3%)
Hematology and oncology	140 (5%)
Cancer	90 (3%)
Renal failure	72 (3%)

## Appendix A

**Table A1:** Alternative drugs of PIM proposed by EU(7) PIM list.

PIM	Alternatives	PIM	Alternatives
aceclofenac	paracetamol	digoxin 0.5 mg	digoxin 0.125 mg
acenocoumarol	warfarin	diltiazem	diltiazem
Acetylsalicylic acid/dipyridamole	Acetylsalicylic acid	dipyridamole	Acetylsalicylic acid
alimemazine	zopiclone 3.75 mg	dipyridamole/Acetylsalicylic acid	Acetylsalicylic acid
alprazolam	oxazepam	Domperidone > 30 mg/day	domperidone 10 mg
amiodarone	amiodarone	dosulepine	sertraline
amitriptyline	sertraline	doxazosine	tamsulosin
aripiprazole	risperidone	escitalopram	sertraline
baclofen	baclofen	esomeprazole	Stop
bromazepam	oxazepam	fesoterodine	Stop
carbamazepine	carbamazepine	flavoxate	Stop
chlorpromazine	risperidone	flecainide	flecainide
citalopram	sertraline	fluoxetine	sertraline
clidinium/chlordiazepoxide	clidinium/ chlordiazepoxide	flurbiprofen	paracetamol
clobazam	oxazepam	fluvoxamine	sertraline
clomipramine	sertraline	glibenclamide	glibenclamide
clonazepam	clonazepam	glimepiride	glimepiride
clorazepate	oxazepam	haloperidol	risperidone
clotiazepam	clotiazepam	hydroxyzine	oxazepam
clozapine	clozapine	ibuprofen	paracetamol
colchicine	colchicine	ibuprofen/codeine	ibuprofen/codeine
colchicine/opium	colchicine/opium	ivabradine	ivabradine
cyamemazine	risperidone	ketoprofen	paracetamol
diazepam	oxazepam	ketoprofen gel	Stop
diclofenac	paracetamol	labetalol	bisoprolol
diclofenac 1% gel	stop	lansoprazole	Stop
diclofenac/misoprostol	diclofenac/misoprostol	levomepromazine	risperidone
loperamide	phloroglucinol	lithium	lithium
loprazolam	zopiclone 3.75 mg	prazepam	oxazepam
lorazepam	lorazepam	prazosin	prazosin
lormetazepam	zopiclone 3.75 mg	propericiazine	risperidone
maprotiline	sertraline	propranolol	bisoprolol
metformin/sitagliptine 1000 mg/50 mg	metformin/sitagliptine 1000 mg/50 mg	rabeprazole	Stop
metformine/vildagliptine 1000 mg/50 mg	metformine/vildagliptine 1000 mg/50 mg	racecadotril	phloroglucinol
metoclopramide	domperidone	ranitidine	Stop
metopimazine	domperidone	rilmenidine	rilmenidine
moxonidine	Stop	risperidone	risperidone
naftidrofuryl	Stop	ropinirole	ropinirole
naproxen	naproxen	rotigotine	rotigotine
nicardipine	nicardipine	scopolamine	domperidone
nifedipine	nifedipine	sitagliptine	sitagliptine
nitrazepam	zopiclone 3.75 mg	sitagliptine/metformin	sitagliptine/metformin
nordazepam	oxazepam	solifenacin	Stop
olanzapine > 10 mg/j	olanzapine 5 mg	sotalol	bisoprolol
omeprazole	Stop	spironolactone	spironolactone
oxazepam > 60 mg/j	oxazepam < 50 mg	spironolactone/altizide	spironolactone/altizide
oxybutynine	Stop	sulindac	Stop
pantoprazole	Stop	terazosine	tamsulosin
paracetamol/codeine	paracetamol/codeine	tramadol	tramadol
paroxetine	sertraline	tramadol/paracetamol	tramadol/paracetamol
phenobarbital	phenobarbital	trihexyphenidyl	Stop
pimozide	risperidone	trimebutine	Stop
pinaverium	Stop	tropatepine	tropatepine
pindolol	bisoprolol	trospium	Stop
pipotiazine	risperidone	venlafaxine	venlafaxine
piracetam	piracetam	verapamil	verapamil
piribedil	piribedil	verapamil/trandolapril	verapamil/trandolapril
piroxicam	piroxicam	vildagliptine	vildagliptine
pramipexole	pramipexole	vildagliptine/metformin	vildagliptine/metformin
zolpidem 10mg > 5 mg/j	zolpidem 10 mg
zopiclone 7.5 mg	zopiclone 3.75 mg
zuclopenthixol	risperidone
